# Characterization of a Novel OTU-like Deubiquitinase from *Babesia microti*: Implications for Babesiosis Treatment

**DOI:** 10.3390/biom16060819

**Published:** 2026-06-01

**Authors:** Betül Yusuf, Fatih Kocabaş

**Affiliations:** 1 College of Engineering, American University of Sharjah, University City, Sharjah 26666, United Arab Emirates; g00101967@aus.edu; 2 Department of Molecular Biology and Genetics, Faculty of Engineering and Natural Sciences, Istanbul Atlas University, Istanbul 34403, Türkiye

**Keywords:** *Babesia microti*, babesiosis, OTU protease, deubiquitinase, apoptosis, immune response

## Abstract

*Babesia microti* is the primary agent of human babesiosis, an emerging tick-borne disease with limited treatment options and growing evidence of drug resistance. Deubiquitinases (DUBs) play critical roles in protein homeostasis and host–pathogen interactions, yet none have been characterized in *B. microti*. Here, we report the first molecular cloning, expression, and functional characterization of an OTU-like cysteine protease from *B. microti* (bm-OTU). The recombinant bm-OTU protein (~22 kDa) was expressed in *E. coli*, purified to high homogeneity, and exhibited ~93% solubility under native conditions. In vitro fluorogenic assays confirmed its deubiquitinase activity. Expression of bm-OTU in HEK293T cells was associated with reduced ubiquitination in cells and increased apoptosis in this overexpression model, as demonstrated by flow cytometry and Western blot analyses. Furthermore, transcriptomic analysis revealed that bm-OTU modulates host immune pathways, notably suppressing the expression of interferon-stimulated genes (*APOBEC3G*, *G1P2*) while upregulating the pro-apoptotic gene *BAK* and the inflammasome sensor *AIM2*. These findings establish bm-OTU as a functional deubiquitinase and a potential virulence factor that may contribute to immune evasion and pathogenesis in babesiosis and may represent a potential target, pending further validation.

## 1. Introduction

Human babesiosis is an emerging tick-borne zoonotic disease caused by protozoan parasites of the genus *Babesia*, with *Babesia microti* being the primary causative agent in North America and Europe [1,2]. The disease is transmitted primarily through the bite of infected *Ixodes scapularis* ticks and, less commonly, via blood transfusion or transplacental transmission [3]. Clinical manifestations range from asymptomatic infection to mild-to-moderate influenza-like illness (fever, chills, myalgia, fatigue) and severe, life-threatening disease characterized by multi-organ failure, acute respiratory distress, and hemolytic anemia, particularly in immunocompromised individuals, the elderly, and asplenic patients [4,5].

Current treatment regimens for babesiosis include antimicrobial combinations, primarily atovaquone plus azithromycin for mild-to-moderate cases and clindamycin plus quinine for severe infections [6]. However, treatment failure and relapse have been increasingly reported, especially in immunocompromised patients [7], and drug resistance is emerging [8]. These challenges underscore the urgent need for novel therapeutic strategies targeting essential parasite pathways.

The ubiquitin-proteasome pathway (UPP) is a critical regulator of protein homeostasis in all eukaryotes, including apicomplexan parasites [9]. The UPP involves sequential activation of ubiquitin by E1-activating enzymes, conjugation by E2 enzymes, and ligation by E3 ligases to target proteins, which are subsequently degraded by the 26S proteasome [10]. Deubiquitinases (DUBs) reverse this process by removing ubiquitin moieties from target proteins, thereby regulating protein stability, signaling, and cellular responses [11].

The ovarian tumor domain (OTU) family of DUBs includes both eukaryotic and viral members. Notably, the *Crimean-Congo hemorrhagic fever virus* (CCHFV) and *Plasmodium* species encode an OTU protease that exhibits deubiquitinase activity and cleaves the ubiquitin-like protein ISG15, thereby antagonizing host innate immune responses and facilitating viral invasion [12,13]. Structural studies have identified critical catalytic pockets (Y89-W99) in the CCHFV OTU protease that are promising targets for small-molecule inhibitor development [14].

Bioinformatic analyses have revealed that *B. microti* encodes an uncharacterized OTU-like cysteine protease (bm-OTU; accession number CCF72503.1). Given the functional similarity between apicomplexan parasites, CCHFV and *Plasmodium* in their OTU protease domains [15], we hypothesized that bm-OTU possesses deubiquitinase activity that may contribute to parasite survival and host immune evasion. Because OTU-family deubiquitinases from other pathogens modulate cell death pathways, we also examined whether bm-OTU expression affects host cell survival and immune gene expression.

In this study, we report the first molecular cloning, expression, purification, and functional characterization of the bm-OTU protein. We demonstrate its deubiquitinase activity in vitro and its effects on intracellular ubiquitination, apoptosis, and cellular immune pathways in mammalian cells. These findings establish bm-OTU as a potential therapeutic target for babesiosis.

## 2. Materials and Methods

### 2.1. Plasmid Constructs and Cloning

The *B. microti* OTU-like protein gene (accession number CCF72503.1) was cloned into the pET26b(+) bacterial expression vector (Novagen, Madison, WI, USA) with a C-terminal 6×His tag. For mammalian expression, the bm-OTU gene was excised from pET26b(+) using the XbaI (NheI-compatible) and EcoRI restriction enzymes (New England Biolabs, Ipswich, MA, USA) and subcloned into the pcDNA3.1(+) mammalian expression vector (Invitrogen, Carlsbad, CA, USA) digested with NheI and EcoRI. Ligation was performed using T4 DNA ligase (NEB) with a 3:1 insert:vector ratio. The recombinant plasmid was transformed into *E. coli* DH5α competent cells and selected on ampicillin-containing LB agar plates. All constructs were confirmed by restriction digestion and DNA sequencing.

### 2.2. Recombinant Protein Expression and Purification

*E. coli* BL21(DE3) competent cells harboring pET26b(+)-bm-OTU were grown in LB broth containing 50 μg/mL kanamycin at 37 °C with shaking (180 rpm) until OD600 reached 0.6. Protein expression was induced with 1 mM isopropyl β-D-1-thiogalactopyranoside (IPTG), and cells were incubated overnight at 24 °C. Cells were harvested by centrifugation (6000 rpm, 10 min, 15 °C), resuspended in lysis buffer (8 M urea, 20 mM Tris-HCl, pH 8.0), and disrupted by sonication for 7 min. The lysate was centrifuged (13,000 rpm, 30 min, 6 °C), and the supernatant was filtered through 0.44 μm syringe filters. The 6×His-tagged bm-OTU protein was purified by immobilized metal affinity chromatography using a 5 mL Ni-NTA agarose column (GE Healthcare, Marlborough, MA, USA). The column was washed with a washing buffer (8 M urea, 20 mM Tris-HCl, pH 8.0), and the protein was eluted with an elution buffer containing increasing imidazole concentrations. Purified fractions were desalted and concentrated using Amicon Ultra centrifugal filters (0.5 mL, 3 kDa cutoff; Merck Millipore, Darmstadt, Germany) with PBS buffer. Protein concentration was determined using the bicinchoninic acid (BCA) assay (Thermo Scientific, Waltham, MA, USA) with bovine serum albumin (BSA) as a standard.

### 2.3. Protein Solubility Assay

PBS buffer was utilized to control the folding conditions of the purified bm-OTU protein. Briefly, 25 μL of purified protein was added to 475 μL of PBS in a 1.5 mL tube. The sample was incubated for one hour at 4 °C and then centrifuged at 14,000 rpm (maximum speed) for five minutes. The recombinant target protein was expected to be in the supernatant. After centrifugation, 475 μL of the supernatant was transferred to a new Eppendorf tube, while the precipitate was resuspended in double distilled water. Both fractions (supernatant and precipitate) were analyzed by SDS-PAGE to assess protein distribution. Protein concentrations in the supernatant and pellet were determined using a NanoDrop spectrophotometer (Wilmington, DE, USA) at A280, and the solubility factor was calculated as: [Supernatant concentration/(Supernatant + Pellet concentration)] × 100.

### 2.4. Concentration Measurement by BCA Assay

To determine the concentration of the purified protein, a bicinchoninic acid (BCA) assay was performed. BSA standard stock was prepared by dissolving BSA powder in PBS at a concentration of 2 mg/mL. Serial dilutions of the BSA stock were prepared in a 96-well plate. The working solution was prepared by mixing solutions A and B at a 100:1 ratio. Then, 5 μL of protein sample was added to 100 μL of working solution in each well. The plate was incubated at 37 °C for 30 min with gentle shaking. Absorbance measurements were carried out at 562 nm using a spectrophotometer.

### 2.5. Deubiquitinase Activity Assay

Deubiquitinase activity of bm-OTU was quantified using the Ubiquitin-AMC substrate (Ub-AMC; Boston Biochem, Cambridge, MA, USA). The reaction mixture contained the reaction buffer (10 mM HEPES, 100 mM NaCl, 2.5 mM DTT), purified bm-OTU protein (50 μg), and 50 μM Ub-AMC in a final volume of 50 μL in a 96-well plate. Fluorescence was measured using a Thermo Scientific Varioskan LUX microplate reader (Vantaa, Finland) with excitation at 345 nm and emission at 445 nm in kinetic mode for 6 h. Controls included reactions without an enzyme or without the substrate.

### 2.6. Cell Culture and Transfection

Complete DMEM medium was prepared by adding 10% fetal bovine serum (FBS) and 1% antibiotics (penicillin-streptomycin) to DMEM medium and warmed to 37 °C. HEK293T(+) cells were thawed at room temperature. The cells were transferred into 5 mL of complete medium and centrifuged at 1000 rpm for 5 min. The pellet was resuspended in 10 mL of complete medium, transferred into a 25T flask, and incubated at 37 °C in a 5% CO_2_ and 95% relative humidity incubator. To maintain healthy cells, passaging was performed whenever confluency reached approximately 80%. The trypsinization method was used for passaging the HEK293T(+) cells. The medium was removed from the flask, 2 mL of trypsin was added, and the cells were incubated for 5 min at 37 °C. Then, 4 mL of complete medium was added, and the cells were centrifuged at 1500 rpm for 5 min.

For transfection, 30 × 10^3^ HEK293T(+) cells were seeded in a six-well plate with 2 mL of high-glucose DMEM supplemented with 10% FBS (no antibiotics added) and incubated at 37 °C overnight. In an Eppendorf tube, a 1:2 ratio (1 mg/mL) of DNA:PEI (polyethylenimine) was added to 200 μL of serum-free DMEM and incubated at room temperature for 15 min. The mixture was added dropwise to the culture medium and incubated for 24 h. The medium was then refreshed, and the cells were incubated for an additional 72 h. Cells were harvested by the trypsinization method for downstream applications. Empty pcDNA3.1(+) vector was used as a negative control.

### 2.7. Apoptosis Assay by Flow Cytometry

Transfected HEK293T cells were harvested by trypsinization, washed with PBS, and resuspended in a 1× binding buffer. Cells were stained with Annexin V-FITC and propidium iodide (PI) according to the manufacturer’s instructions (BD Biosciences, San Jose, CA, USA). Stained cells were analyzed using a flow cytometer (BD FACSCalibur, San Jose, CA, USA) to quantify early apoptosis (Annexin V+/PI−), late apoptosis (Annexin V+/PI+), and necrosis (Annexin V−/PI+).

### 2.8. Western Blot Analysis

Western blot was performed to identify the intracellular deubiquitinase activity of the recombinant protein from cell lysates containing pcDNA3.1 (empty vector control) and pcDNA3.1-bm-OTU plasmids. Transfected HEK293T(+) cells were harvested for Western blotting.

Sample preparation: The transfected HEK293T(+) cells were harvested, washed with sterile PBS, and centrifuged for five minutes at 1500 rpm. The cell pellet was treated with a RIPA buffer (50 mM Tris-HCl pH 7.4, 150 mM NaCl, 1% NP-40, 0.5% sodium deoxycholate, 0.1% SDS, protease inhibitor cocktail) for 30 min on ice. The cell lysate mixture was then centrifuged, and the supernatant was collected for analysis.

SDS-PAGE: Proteins were separated based on their molecular weight using 12% polyacrylamide gel. Protein concentrations were determined by BCA assay, and 25 μg of each protein sample was mixed with 4× loading buffer and denatured by boiling for five minutes at 95 °C. Then, 20 μL of each sample was loaded per lane into the wells of the polyacrylamide gel. Electrophoresis was run at 100 V for 120 min.

Protein transfer: After SDS-PAGE was completed, proteins were transferred from the polyacrylamide gel onto a PVDF membrane. The membrane, filter papers, and sponges were saturated in a 1× transfer buffer for 5–10 min during assembly of the transfer sandwich for wet transfer. The transfer was run for one and a half hours at 100 V and 400 mA in an iced tank.

Membrane blocking: After electrophoresis was completed, the transfer stack was disassembled. The membrane was incubated for 1 h in a blocking buffer (5% non-fat milk solution in TBST) at room temperature to reduce or eliminate non-specific protein binding.

Primary and secondary antibody staining: The mono- and poly-ubiquitinylated conjugates monoclonal antibody (Enzo Life Sciences, Farmingdale, NY, USA, BML-PW8810-0500) was used as the primary antibody. The primary antibody solution (1:1000 dilution in blocking buffer) was added to the PVDF membrane and incubated at 4 °C overnight with gentle rocking. The membrane was then washed with 1× TBST (Tris-buffered saline with 0.1% Tween 20) five times for 5 min each with gentle rocking to eliminate non-specific background. Subsequently, the membrane was incubated with HRP-conjugated anti-mouse secondary antibody (1:5000 dilution) at room temperature for 60 min with gentle rocking. Finally, the membrane was washed several times in a 1× TBST buffer. After incubating the membrane with enhanced chemiluminescence (ECL) substrate according to the manufacturer’s instructions, the chemiluminescence imaging system (ChemiDoc system, Hercules, CA, USA) was used to expose the membrane and detect the desired proteins. Band intensities were quantified using ImageJ software (1.54t). β-Actin (mouse anti-β-actin; Sigma-Aldrich, St. Louis, MO, USA; 1:5000) was used as a loading control.

### 2.9. RNA Isolation and Real-Time PCR

Total RNA was extracted from transfected HEK293T cells using an RNA isolation kit (Macherey-Nagel, Düren, Germany, 740406.05). cDNA was synthesized using the ProtoScript II First Strand cDNA Synthesis Kit (NEB, Ipswich, MA, USA, Cat No: E6560). Real-time PCR was performed in technical triplicates using the PowerUp SYBR Green Master Mix (Applied Biosystems, Foster City, CA, USA) on a LightCycler 96 system (Roche, Basel, Switzerland). Primer sequences for target genes (*AIM2*, *IL18*, *NFKB1*, *IFNA1*, *IFNA2*, *IFNAR1*, *IFNB1*, *STAT1*, *APOBEC3G*, *G1P2*, *IL15*, *BCL2L1*, *BCL2A1*, *BAK*, *BID*) were obtained from the NIH human primer database (Table 1). *β-Actin* was used as an internal control. Relative gene expression was calculated using the ΔΔCt method.

### 2.10. Statistical Analysis

Data are presented as the mean ± standard error (SE) from at least three independent experiments. Statistical comparisons were performed using Student’s *t*-test. A *p*-value < 0.05 was considered statistically significant.

## 3. Results

### 3.1. Expression and Purification of Recombinant bm-OTU Protein

**Figure 1:**
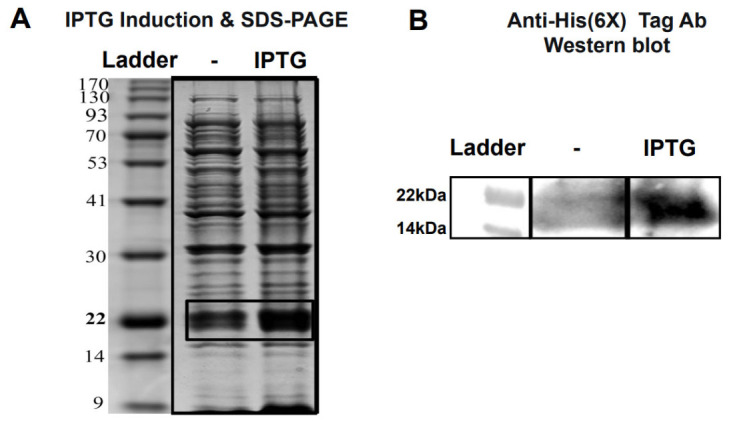
Recombinant expression of bm-OTU protein. (**A**) SDS-PAGE analysis of bm-OTU expression in *E. coli* BL21 cells. -: uninduced (no IPTG); IPTG: induced with 1 mM IPTG. The box indicates the expected 22 kDa bm-OTU band. (**B**) Western blot confirmation of His-tagged bm-OTU expression using anti-His antibody.

The bm-OTU gene (576 bp encoding 192 amino acids) was successfully expressed in *E. coli* BL21(DE3) cells under IPTG induction. SDS-PAGE analysis revealed a prominent protein band at approximately 22 kDa, consistent with the predicted molecular mass, in IPTG-induced cell lysates but not in uninduced controls (Figure 1A). Western blot analysis using the anti-His-tag antibody confirmed the expression of His-tagged bm-OTU protein (Figure 1B). A faint band of similar size in the uninduced control likely reflects basal (leaky) expression, a known phenomenon in BL21(DE3) cells. The smeared appearance of the induced band may indicate slight protein degradation or incomplete denaturation.

**Figure 2:**
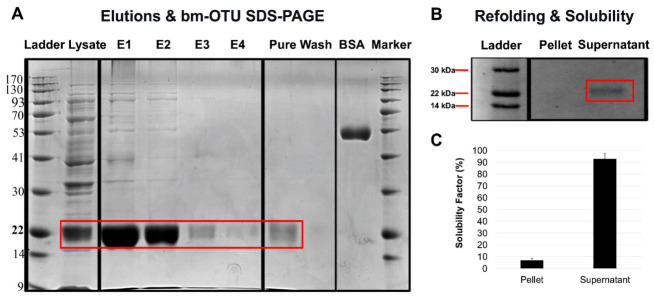
Purification and solubility analysis of bm-OTU. (**A**) SDS-PAGE analysis of Ni-NTA purification lysate, elution fractions (E1–E4), high purity (Pure), flow-through, wash, BSA. (**B**) SDS-PAGE showing supernatant and pellet fractions after centrifugation in PBS. (**C**) Quantitative solubility factor (bar graph) showing approximately 93% solubility in PBS. Red boxes highlight bm-OTU proteins.

Purification of bm-OTU by Ni-NTA affinity chromatography yielded four elution fractions (E1–E4), with E1 and E2 containing the highest protein concentrations (Figure 2A). Ultra-purification using Amicon columns resulted in a single band of purified bm-OTU. The BCA assay standard curve gave an average protein concentration of 1.21 mg/mL for the purified bm-OTU (Table 2). An uninduced control was not included in the purification gel; however, the presence of a single prominent band in elution fractions (E1–E4) and the pure (P) lane is consistent with successful purification.

### 3.2. Solubility of Recombinant bm-OTU Protein

To assess proper protein folding, the solubility of purified bm-OTU was evaluated in a PBS buffer. SDS-PAGE analysis showed that the majority of bm-OTU protein was present in the supernatant fraction after centrifugation, with minimal protein in the pellet (Figure 2B). Quantitative analysis using NanoDrop measurements revealed a solubility factor of approximately 93% (Figure 2C), indicating that the recombinant protein was predominantly soluble and properly folded under native conditions.

### 3.3. Deubiquitinase Activity of bm-OTU

**Figure 3:**
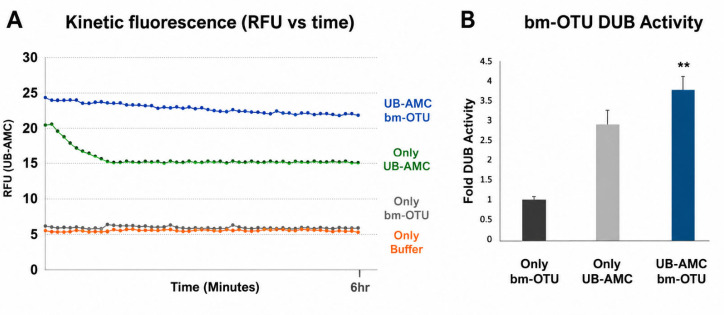
In vitro deubiquitinase activity of bm-OTU measured by Ub-AMC fluorescence assay. (**A**) Kinetic fluorescence measurements over 6 h showing relative fluorescence units (RFU) for bm-OTU + Ub-AMC, Ub-AMC only, bm-OTU only, and buffer only. (**B**) Statistical comparison of deubiquitinase activity between bm-OTU and control. ** *p* < 0.01.

The deubiquitinase activity of purified bm-OTU was assessed using the Ub-AMC fluorogenic substrate. Cleavage of Ub-AMC by deubiquitinase activity results in release of free AMC, which can be quantified by fluorescence. Kinetic measurements over 6 h showed that bm-OTU exhibited detectable deubiquitinase activity compared to the substrate-only control (Figure 3A). However, the activity was modest and did not increase progressively over time, starting instead from a relatively high baseline. Statistical analysis revealed a significant difference between bm-OTU and control groups (*p* < 0.01) (Figure 3B). These results confirm that bm-OTU possesses deubiquitinase activity, although optimization of assay conditions may be required to enhance observed activity.

### 3.4. bm-OTU Induces Apoptosis in HEK293T Cells

**Figure 4:**
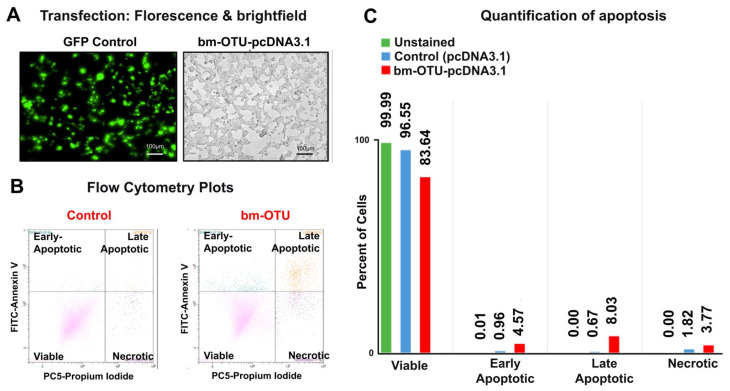
Transfection and apoptosis analysis. (**A**) Representative microscopy images showing GFP transfection control (**left**) and bm-OTU-pcDNA3.1-transfected HEK293T cells (**right**). (**B**) Flow cytometry plots of bm-OTU-pcDNA3.1-transfected cells compared to pcDNA3.1-transfected cells. (**C**) Flow cytometry apoptosis analysis (Annexin V/PI staining) showing quantification of live, early apoptotic, late apoptotic, and necrotic cells for unstained, pcDNA3.1 control and bm-OTU-pcDNA3.1 transfection.

The bm-OTU gene was successfully subcloned from pET26b(+) into the pcDNA3.1(+) mammalian expression vector. The constructed bm-OTU-pcDNA3.1 plasmid was transfected into HEK293T cells using PEI, with GFP used as a transfection control (Figure 4A). Microscopic analysis revealed over 65% transfection efficiency.

The effect of bm-OTU expression on apoptosis was evaluated by flow cytometry using Annexin V/PI staining (Figure 4B). Compared to the pcDNA3.1 empty vector control, bm-OTU-expressing cells showed a significant increase in early apoptosis (Annexin V+/PI−), late apoptosis (Annexin V+/PI+), and necrosis (Annexin V−/PI+) (Figure 4C). These results indicate that bm-OTU expression promotes cell death through apoptotic and necrotic pathways.

### 3.5. bm-OTU Reduces Intracellular Ubiquitination

**Figure 5:**
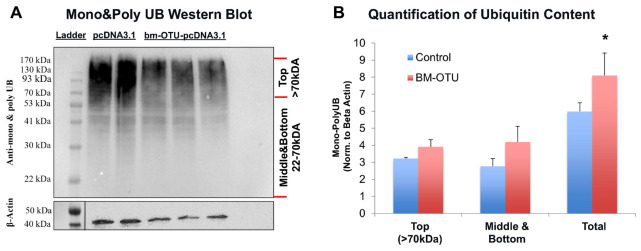
Effects of bm-OTU on intracellular ubiquitination. (**A**) Western blot analysis using mono-/poly-ubiquitin antibody showing ubiquitinylated protein smears in control (pcDNA3.1) and bm-OTU-expressing cells. β-Actin is shown as a loading control. The blot is divided into two major regions: top (>70 kDa) and middle bottom (22–70 kDa). Original western blots can be found at Supplementary Materials. (**B**) Quantitative analysis of ubiquitinylation levels in the two regions along with total ubiquitination normalized to control. * *p* < 0.05.

To investigate the intracellular deubiquitinase activity of bm-OTU, Western blot analysis was performed on lysates from transfected HEK293T cells using an antibody that recognizes both mono- and poly-ubiquitinylated conjugates. The antibody detects ubiquitin-protein conjugates but not free ubiquitin. Results showed smear bands characteristic of ubiquitinylated proteins in both control and bm-OTU-expressing cells (Figure 5A). However, quantification of band intensities revealed significantly reduced ubiquitinylation in bm-OTU-expressing cells compared to controls (Figure 5B, *p* < 0.05 for total/all regions). β-Actin loading control confirmed equal protein loading. These findings demonstrate that bm-OTU functions as a deubiquitinase in vivo, reducing global intracellular ubiquitination levels.

### 3.6. bm-OTU Modulates Cellular Immune Pathway Gene Expression

**Figure 6:**
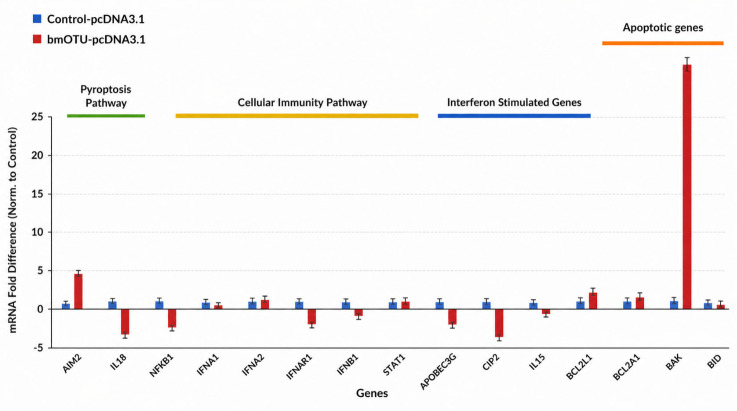
Effects of bm-OTU on cellular immunity and apoptotic gene expression. RT-qPCR analysis of selected genes involved in pyroptosis (*AIM2*, *IL18*), NF-κB signaling (NFKB1), type I interferon signaling (*IFNA1*, *IFNA2*, *IFNAR1*, *IFNB1*), interferon-stimulated genes (*STAT1*, *APOBEC3G*, *G1P2*, *IL15*), and apoptosis (*BCL2L1*, *BCL2A1*, *BAK*, *BID*) were analysis. Gene expression levels were normalized to *β-actin* and are shown relative to pcDNA3.1 control (set to 1).

The effect of bm-OTU expression on host cellular immune responses was assessed by RT-qPCR analysis of selected genes involved in innate immunity, interferon signaling, and apoptosis (Figure 6). Key findings indicate that the pyroptosis pathway is activated in bm-OTU-expressing cells, as evidenced by the upregulation of AIM2, a cytosolic dsDNA sensor, compared to controls. In contrast, the NF-κB pathway appears to be slightly suppressed, with *NFKB1* and *INFRA1* showing a modest decrease in expression. The type I interferon pathway exhibited limited changes, with IFNAR1 modestly downregulated while *IFNA1*, *IFNA2*, and *IFNB1* remained largely unchanged. Among interferon-stimulated genes (ISGs), a more pronounced reduction was observed in *APOBEC3G* and *G1P2*. Regarding apoptosis-related genes, *BCL2L1*, *BCL2A1*, and *BID* showed increased expression with no significant changes; however, the pro-apoptotic gene *BAK* was markedly upregulated.

These results indicate that bm-OTU selectively modulates host gene expression, particularly suppressing ISG expression while upregulating pro-inflammatory and pro-apoptotic mediators.

## 4. Discussion

This study presents the first molecular and functional characterization of the OTU-like deubiquitinase from *Babesia microti* (bm-OTU). The identification and characterization of this protein fill a critical gap in our understanding of babesiosis pathogenesis and identify a potential new therapeutic target.

The successful expression and purification of recombinant bm-OTU from *E. coli* yielded a soluble protein of the expected molecular mass (22 kDa) with high purity. The 93% solubility in PBS suggests proper folding under native conditions, which is essential for functional studies [16]. Protein misfolding and aggregation are common challenges in recombinant protein production, and our optimized conditions (overnight induction at 24 °C and urea-based lysis followed by refolding in PBS) appear to have minimized these issues.

The deubiquitinase activity of bm-OTU was confirmed using the Ub-AMC fluorogenic assay, although the observed activity was modest. Several factors may account for this: Suboptimal folding of the catalytic domain in the bacterial expression system, despite overall solubility; Absence of cofactors or post-translational modifications required for full activity; Assay conditions (pH, temperature, buffer composition) may require optimization for this particular OTU family member. Previous studies on CCHFV OTU protease demonstrated robust activity under similar conditions [14], suggesting that bm-OTU may have intrinsically lower catalytic efficiency or different substrate specificity. Future studies should test alternative substrates (e.g., ISG15-AMC, di-ubiquitin chains) and optimize assay parameters.

The functional consequences of bm-OTU expression in mammalian cells were striking. Flow cytometry analysis revealed an induction of apoptosis and necrosis in bm-OTU-transfected HEK293T cells. This pro-apoptotic effect is consistent with the known role of deubiquitinases in regulating cell death pathways [17]. Ubiquitination is a key post-translational modification that controls the stability and activity of pro- and anti-apoptotic proteins [18]. By reducing global ubiquitination levels, bm-OTU may destabilize anti-apoptotic factors or activate pro-apoptotic pathways. The dramatic upregulation of *BAK* (a pro-apoptotic BCL2 family member) in bm-OTU-expressing cells supports this mechanism. However, the gene expression changes are based solely on mRNA levels that require protein-level validations (e.g., Western blot for BAK). We also acknowledge that overexpression in HEK293T cells may induce non-physiological responses. The observed apoptosis and gene expression changes could be partially or largely due to supraphysiological levels of a DUB rather than specific bm-OTU functions. A catalytically inactive mutant is required to distinguish direct from indirect effects. Additionally, the natural host cell of *B. microti* (mature erythrocyte) lacks transcription; thus, immune modulation likely occurs in other cell types (e.g., macrophages, endothelial cells). Our findings should be considered hypothesis-generating and validated in more relevant models.

The reduction in global intracellular ubiquitination detected by the mono-poly ubiquitin antibody provides direct evidence that bm-OTU functions as a deubiquitinase in vivo. This finding aligns with our hypothesis that bm-OTU shares functional similarity with viral and malarial OTU proteases, which also exhibit deubiquitinase activity during host cell invasion [12,13]. The effect was observed across all molecular weight ranges, suggesting that bm-OTU acts on multiple ubiquitinylated substrates rather than being substrate-specific.

The modulation of host immune gene expression by bm-OTU reveals potential immune evasion strategies employed by *B. microti*. The downregulation of interferon-stimulated genes (*APOBEC3G*, *G1P2*) is particularly noteworthy, as ISGs are critical components of the antiviral and antiparasitic innate immune response [19]. Suppression of ISG expression by bm-OTU may allow the parasite to evade host defense mechanisms, similar to viral OTU proteases that cleave ISG15 and inhibit interferon signaling [20]. The upregulation of *AIM2* suggests activation of the pyroptosis pathway, a pro-inflammatory form of cell death that may contribute to the immunopathology observed in severe babesiosis [21,22,23,24,25].

The slight decrease in *NFKB1* expression is interesting, as NF-κB is a master regulator of inflammation. CCHFV OTU protease has been shown to antagonize NF-κB signaling [14], and our results suggest bm-OTU may have a similar effect, although the magnitude of change was modest.

This study has several limitations. First, the in vitro deubiquitinase activity was lower than expected, and optimization of assay conditions is warranted. Second, we used HEK293T cells as a model system; future studies should validate these findings in relevant cell types, such as erythrocytes or immune cells. Third, the specific ubiquitin chain linkages (K48, K63, etc.) cleaved by bm-OTU remain unknown. Fourth, identification of direct substrates of bm-OTU would provide mechanistic insight. In addition, HEK293T cells are not physiologically relevant for an intraerythrocytic parasite. Future studies should evaluate bm-OTU effects in immune cells (e.g., THP-1 macrophages) or animal models of babesiosis. Finally, the development of specific small-molecule inhibitors targeting the bm-OTU catalytic pocket (analogous to the Y89-W99 pocket in CCHFV [14]) represents an important future direction.

## 5. Conclusions

In conclusion, this study provides the first characterization of the *Babesia microti* OTU-like deubiquitinase. We successfully expressed, purified, and demonstrated the deubiquitinase activity of this protein in vitro and in vivo. bm-OTU expression in mammalian cells reduced global ubiquitination, induced apoptosis, and modulated host immune gene expression, particularly suppressing interferon-stimulated genes while upregulating pro-inflammatory and pro-apoptotic mediators. These findings establish bm-OTU as a potential virulence factor and therapeutic target for babesiosis. Future work should focus on developing specific bm-OTU inhibitors and validating these results in animal models of babesiosis [26,27,28,29,30].

## Figures and Tables

**Table 1:** List of Primers used in this study.

Gene	Forward Primer (5′–3′)	Reverse Primer (5′–3′)	Role in Cellular Immune Response
*AIM2*	GCAGTGATGAAGACCATTCG	GGCTTTGGTTTTGTTACCGA	Cytosolic dsDNA sensor pathway
*IL18*	TGCAGTCTACACAGCTTCGG	ACTGGTTCAGCAGCCATCTT	Cytosolic dsDNA sensor pathway
*NFKB1*	ATGTATGTGAAGGCCCATCC	ATAACCTTTGCTGGTCCCAC	Antiviral response, cellular immune response pathway
*IFNA1*	CAGAGTCACCCATCTCAGCA	CTTGACTTGCAGCTGAGCAC	Type-I Interferon signal pathway
*IFNA2*	GCTCACCCATTTCAACCAGT	CTTGACTTGCAGCTGAGCAC	Type-I Interferon signal pathway
*IFNAR1*	GACCCTAGTGCTCGTGCGAC	TCATCGCTCCTGTTCCAC	Type-I Interferon signal pathway
*IFNB1*	CTTTCGAAGCCTTTGCTCTG	CAGGAGAGCAATTTGGAGGA	Type-I Interferon signal pathway
*STAT1*	TTCAGGAAGACCCAATCCAG	TGCTCTGAATATTCCCCGAC	Type-I Interferon signal pathway
*APOBEC3G*	AGGGGCTTTCTATGCAACC	TTCCAAAAGGGAATCACGTC	Interferon Stimulated Genes (ISG)
*G1P2*	GCGAACTCATCTTTGCCAGT	AGGGACACCTGGAATTCGTT	Interferon Stimulated Genes (ISG)
*IL15*	AGAAGCCAACTGGGTGAATG	ACTTTGCAACTGGGGTGAAC	Interferon Stimulated Genes (ISG)
*BCL2L1*	GAGCTGGTGGTTGACTTTCTC	TCCATCTCCGATTCAGTCCCT	Apoptotic genes
*BCL2A1*	TACAGGCTGGCTCAGGACTAT	CGCAACATTTTGTAGCACTCTG	Apoptotic genes
*BAK*	GTTTTCCGCAGCTACGTTTTT	GCAGAGGTAAGGTGACCATCTC	Apoptotic genes
*BID*	ATGGACCGTAGCATCCCTCC	GTAGGTGCGTAGGTTCTGGT	Apoptotic genes

**Table 2:** Concentration measurements of purified bm-OTU protein by the BCA assay.

Measurement	Absorbance (562 nm)	Concentration (mg/mL)
1	0.267	1.09
2	0.323	1.33
Average		1.21

## Data Availability

All data generated or analyzed during this study are included in this published article and its Supplementary Materials Files.

## Supplementary Materials

The following supporting information can be downloaded at: https://www.mdpi.com/article/10.3390/biom16060819/s1, Original western blots.
