# Supplementary figures and images for: Characterization of a Novel OTU-like Deubiquitinase from Babesia microti: Implications for Babesiosis Treatment
Source: Biomolecules. 2026 Jun 1;16(6):819. doi: 10.3390/biom16060819 (PMC13296843; doi:10.3390/biom16060819)

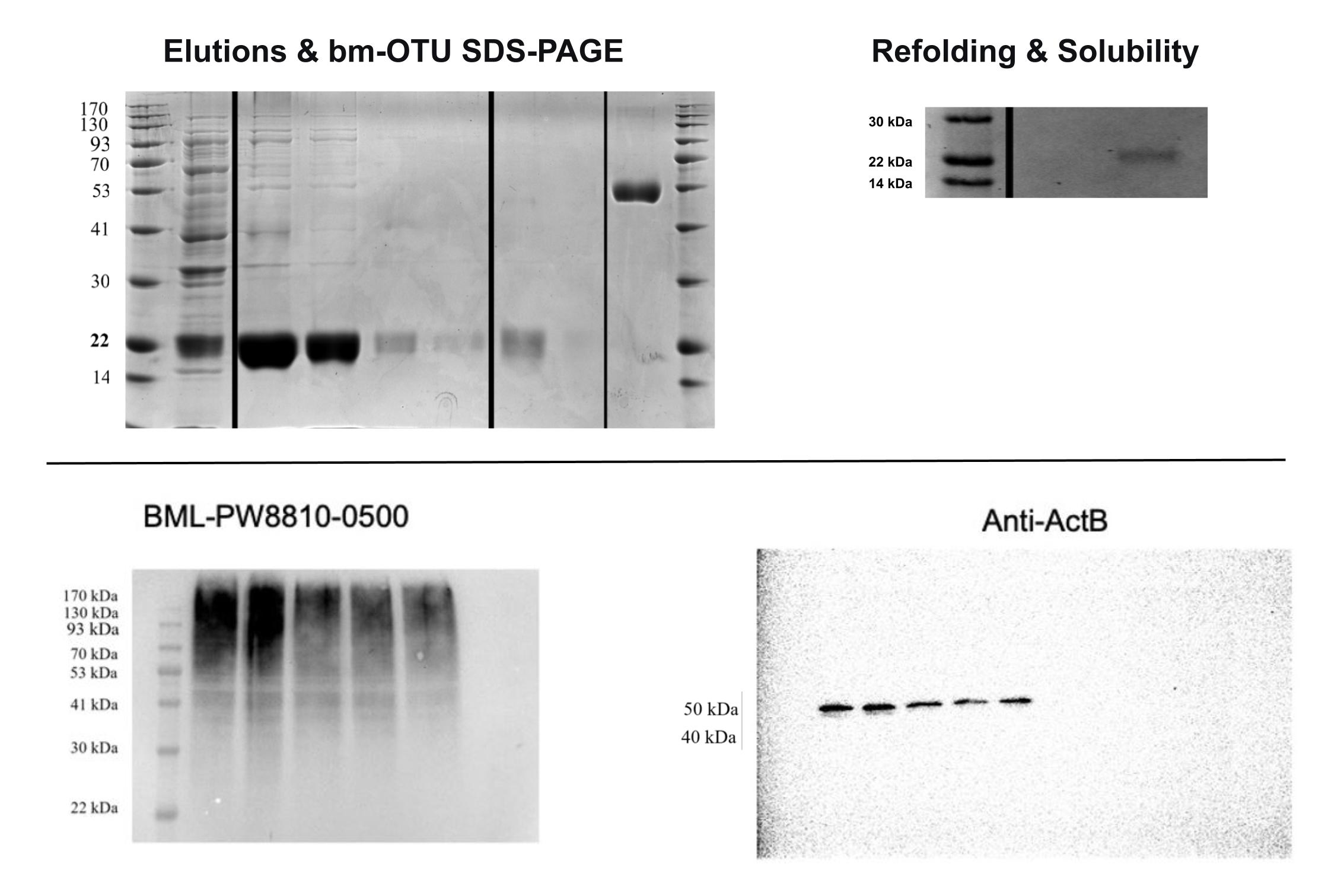

Supplement: Supplementary file 1 [file biomolecules-16-00819-s001.zip › biomolecules-4286772-supplementary.jpg]
